# Global and Spatial Compartmental Interrelationships of Bone Density, Microstructure, Geometry and Biomechanics in the Distal Radius in a Colles’ Fracture Study Using HR-pQCT

**DOI:** 10.3389/fendo.2021.568454

**Published:** 2021-05-26

**Authors:** Kazuteru Shiraishi, Andrew J. Burghardt, Makoto Osaki, Sundeep Khosla, Julio Carballido-Gamio

**Affiliations:** ^1^ Department of Radiology, University of Colorado Anschutz Medical Campus, Aurora, CO, United States; ^2^ Department of Orthopedic Surgery, Nagasaki University Graduate School of Biomedical Sciences, Nagasaki, Japan; ^3^ Department of Radiology and Biomedical Imaging, University of California, San Francisco, San Francisco, CA, United States; ^4^ Division of Endocrinology, Diabetes, Metabolism and Nutrition, Department of Internal Medicine, College of Medicine, Mayo Clinic, Rochester, MN, United States

**Keywords:** Colles’ fracture, HR-pQCT (high-resolution peripheral quantitative computed tomography), bone, interrelationships, spatial analysis

## Abstract

**Background:**

Bone parameters derived from HR-pQCT have been investigated on a parameter-by-parameter basis for different clinical conditions. However, little is known regarding the interrelationships of bone parameters and the spatial distribution of these interrelationships. In this work: 1) we investigate compartmental interrelationships of bone parameters; 2) assess the spatial distribution of interrelationships of bone parameters; and 3) compare interrelationships of bone parameters between postmenopausal women with and without a recent Colles’ fracture.

**Methods:**

Images from the unaffected radius in fracture cases (n=84), and from the non-dominant radius of controls (n=98) were obtained using HR-pQCT. Trabecular voxel-based maps of local bone volume fraction (L.Tb.BV/TV), homogenized volumetric bone mineral density (H.Tb.BMD), homogenized μFEA-derived strain energy density (H.Tb.SED), and homogenized inter-trabecular distances (H.Tb.1/N) were generated; as well as surface-based maps of apparent cortical bone thickness (Surf.app.Ct.Th), porosity-weighted cortical bone thickness (Surf.Ct.SIT), mean cortical BMD (Surf.Ct.BMD), and mean cortical SED (Surf.Ct.SED). Anatomical correspondences across the parametric maps in the study were established *via* spatial normalization to a common template. Mean values of the parametric maps before spatial normalization were used to assess compartmental Spearman’s rank partial correlations of bone parameters (e.g., between H.Tb.BMD and L.Tb.BV/TV or between Surf.Ct.BMD and Surf.app.Ct.Th). Spearman’s rank partial correlations were also assessed for each voxel and vertex of the spatially normalized parametric maps, thus generating maps of Spearman’s rank partial correlation coefficients. Correlations were performed independently within each group, and compared between groups using the Fisher’s Z transformation.

**Results:**

All within-group global trabecular and cortical Spearman’s rank partial correlations were significant; and the correlations of H.Tb.BMD–L.Tb.BV/TV, H.Tb.BMD–H.Tb.1/N, L.Tb.BV/TV–H.Tb.1/N, Surf.Ct.BMD–Surf.Ct.SED and Surf.Ct.SIT–Surf.Ct.SED were significantly different between controls and fracture cases. The spatial analyses revealed significant heterogeneous voxel- and surface-based correlation coefficient maps across the distal radius for both groups; and the correlation maps of H.Tb.BMD–L.Tb.BV/TV, H.Tb.BMD–H.Tb.1/N, L.Tb.BV/TV–H.Tb.1/N, H.Tb.1/N–H.Tb.SED and Surf.app.Ct.Th - Surf.Ct.SIT yielded small clusters of significant correlation differences between groups.

**Discussion:**

The heterogeneous spatial distribution of interrelationships of bone parameters assessing density, microstructure, geometry and biomechanics, along with their global and local differences between controls and fracture cases, may help us further understand different bone mechanisms of bone fracture.

## Introduction

Osteoporosis is defined as a skeletal disorder characterized by compromised bone strength predisposing a person to an increased risk of fracture (1). Bone strength depends on both bone mineral density (BMD) and bone quality, which includes factors such as bone microstructure, micro fracture, bone turnover, and mineralization (1). Therefore, areal BMD (aBMD) derived from dual energy X-ray absorptiometry (DXA), which is the clinical standard for osteoporosis assessment, provides a limited evaluation of bone strength (2). This limitation has been manifested with non-osteoporotic subjects (T-score > -2.5) sustaining fragility fractures (3, 4). Consequently, additional assessments such as volumetric BMD (BMD) and quantification of the trabecular and cortical bone microstructure and geometry, might improve the prevention of fragility fractures (5–8).

High-resolution peripheral quantitative computed tomography (HR-pQCT) enables *in vivo* visualization of the three-dimensional (3D) bone microstructure at the distal radius and tibia with high spatial resolution and a low level of radiation exposure (9, 10). Because of its small voxel size (82 μm isotropic; 61 μm isotropic in the second-generation scanner), this unique medical imaging modality can distinguish between cortical and trabecular bone, and allows 3D assessments of microstructure, density, and geometry. In addition, HR-pQCT provides the capability of estimating bone strength using micro-finite element analysis (μFEA) (11, 12). Since the introduction of this high-resolution imaging modality, many studies have been performed to evaluate the effectiveness of several bone microstructural features in the assessment of various bone disorders (13).

Colles’ fracture is the most common fragility fracture in the distal radius, with osteoporosis being a risk factor, especially for postmenopausal women (14). Existing studies using HR-pQCT have demonstrated that lower BMD and deterioration of bone microstructure and geometry in the cortical and trabecular bone compartments are associated with fragility fractures in postmenopausal women (5–7, 11, 15–17), particularly for wrist fracture (12, 16, 18, 19). However, although it is known that density, quality of bone microstructure, geometry, and bone strength estimates derived from μFEA are significantly correlated (15), it is unknown how these associations are affected in subjects sustaining fragility fractures. Furthermore, it is unknown if these associations are spatially homogeneous, even in healthy subjects. We hypothesize than in subjects with a recent Colles’ fracture, there is a disruption in the interrelationship of bone parameters. The purpose of this work was then to investigate interrelationships of bone parameters in postmenopausal women with and without a recent Colles’ fracture to help our understanding of potential mechanisms of bone fracture using HR-pQCT. In particular, interrelationships of density, microstructural, geometrical and biomechanical parameters derived from μFEA were investigated globally and spatially for both the trabecular and cortical bone compartments.

## Methods

### Subjects

This work was based on existing data from the study of Melton et al. (18). Recruited subjects were 100 postmenopausal women newly diagnosed with a Colles’ fracture in Olmsted County, Minnesota; and 105 postmenopausal controls frequency-matched based on the expected age distribution of forearm fractures in that community. The event that precipitated the fracture was characterized according to the scheme of Palvanen and colleagues (20). Controls had no history of another osteoporotic fracture that occurred after age 35 years.

This research was conducted in accordance to the regulations of the participating institutions and informed consent was obtained from all participants prior to enrollment. Analyses were performed based on de-identified data.

### Imaging

**Figure 1:**
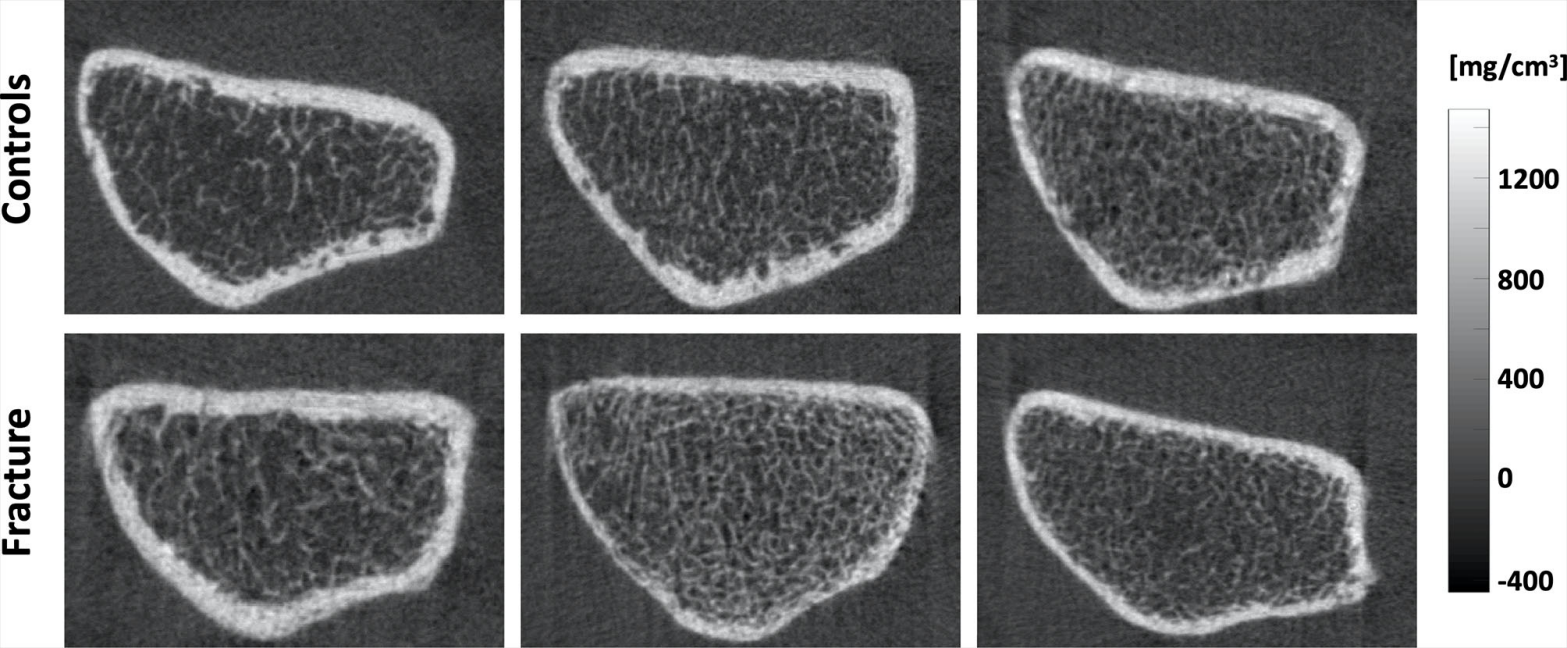
Representative axial cross-sections of HR-pQCT images of the distal radius for three controls (top) and three fracture cases (bottom).

Imaging of the distal radius for each subject was performed using an HR-pQCT system (XtremeCT; Scanco Medical AG, Brüttisellen, Switzerland). The forearm was fixed in a carbon fiber cast, then inserted into the gantry of the scanner. The non-dominant side in controls and the unaffected side in fracture cases was scanned. The starting point of the fixed scan region was 9.5 mm proximal from a reference line set manually at the endplate in the distal radius. Scan settings were as follows: voltage 60 kVp, current 900 μA, integration time 100 ms, and effective dose 4.2 μSv. A 9.02 mm section spanned by a total of 110 slices was imaged with an isotropic voxel size of 82 μm. Subjects were rescanned if clear images were not obtained. For this study, all scans were scored for presence and severity of motion artifacts using the current artifact grading scheme of the manufacturer (21). Grades are based on the appearance of horizontal streakings, contiguity of cortical bone, and amount of trabecular smearing. Grade 1 represents no motion artifacts, Grade 2 minor motion artifacts, Grade 3 moderate motion artifacts, and Grades 4 and 5 represent severe and extreme motion artifacts, respectively. Only scans with image quality scores of 1 to 3 were analyzed in this work. **Figure 1** shows representative axial cross-sections of HR-pQCT scans of the distal radius for three controls and three fracture cases.

### Image Analysis

#### Standard Analysis

Bone segmentation from HR-pQCT images was performed using the standard evaluation software provided by the manufacturer as mentioned in other studies (22, 23). Briefly, the periosteal contours of the distal radius were identified semi-automatically using an edge-finding algorithm, checked visually, and modified manually as necessary (24). Then, a threshold-based algorithm was used to segment the cortical and trabecular bone compartments enabling compartment-specific assessments of density, microstructure, geometry, and biomechanics (25).

The following parameters were measured for the trabecular and cortical bone compartments: trabecular BMD (Tb.BMD), trabecular bone volume fraction (Tb.BV/TV), trabecular number (Tb.N), trabecular separation (Tb.Sp), cortical BMD (Ct.BMD), and cortical thickness (Ct.Th) (26).

In addition, assessment of apparent biomechanical parameters was accomplished using linear μFEA as previously described in the literature (27). Briefly, a mesh of isotropic elements was generated from the voxel-based representation of trabecular and cortical bone. Elements in this mesh were assigned an elastic modulus of 6.829 GPa and a Poisson ratio of 0.3. Using the iterative solver provided by the manufacturer, reaction forces on the superior and inferior ends of the model were then calculated for a 1% axial compression. Based on these estimates, failure load (μFEA.FL) and bone stiffness (μFEA.Stiffness) were computed as in Mueller et al. (27).

#### Spatial Analysis

To incorporate parameters assessing bone microstructure and biomechanics in our spatial analyses, we generated maps representing inter-trabecular distances (Tb.1/N) and μFEA-derived strain energy density (SED; J/mm^3^). SED is defined as the potential energy stored in a volume by virtue of an elastic deformation (18). Then to obtain smooth maps of bone parameters suitable for voxel-wise associations at the population level, we generated homogenized maps of BMD, Tb.1/N and SED, as well as maps representing local assessments of bone volume fraction using spherical kernels (*r*=11 for BMD, SED and BV/TV; *r*=5 for Tb.1/N) (28).

**Figure 2:**
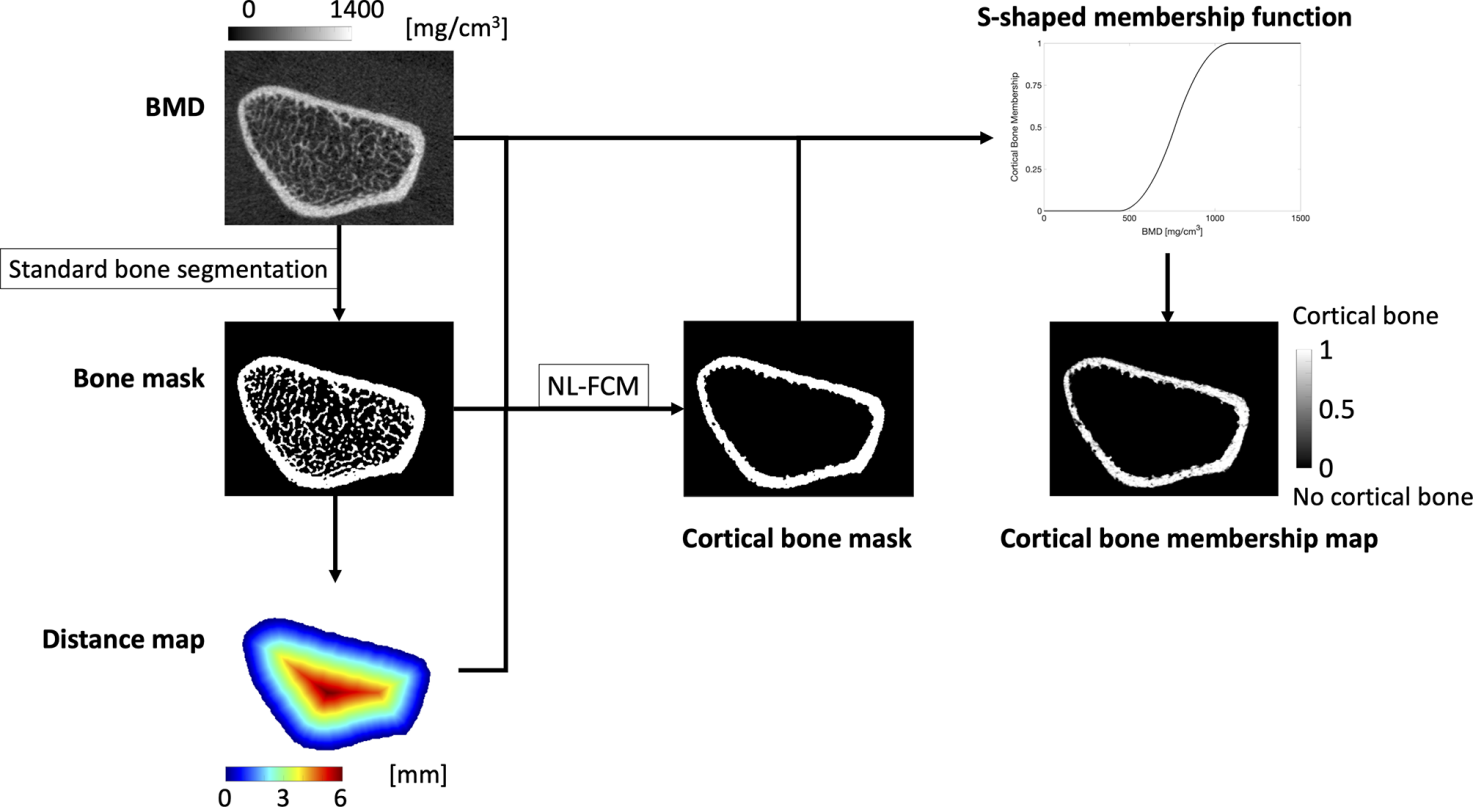
The standard bone segmentation provided by the manufacturer was used to generate a bone mask. The periosteal surface was then used to compute a distance map, that together with the BMD map and the bone mask was fed to a non-local fuzzy c-means clustering algorithm (NL-FCM) to identify the endosteal surface. BMD values within the cortex were used to generate a s-shaped membership function that mapped each BMD value to a value between 0 and 1, indicating the degree of membership of each voxel to the category of cortical bone.

Periosteal surface-based maps encoding the local apparent cortical bone thickness (Surf.app.Ct.Th), effective cortical bone thickness taking into account porosity and partial volume effects (Surf.Ct.SIT; streamline integral thickness), mean cortical BMD (Surf.Ct.BMD), and mean cortical SED (Surf.Ct.SED) at each vertex were also generated as previously described by Carballido-Gamio et al. enabling population-based vertex-wise associations of cortical bone parameters (28). For this purpose, the cortical compartment was identified with an in-house implementation of a non-local fuzzy c-means (NL-FCM) algorithm using BMD maps, bone segmentations, and distances to the periosteal surfaces as clustering features (28). Then, soft cortical bone classification was performed using a fuzzy s-shaped membership function assigning to each voxel a value between 0 (no cortical bone) and 1 (cortical bone) (29), indicating the degree of membership of a voxel to the category of cortical bone (**Figure 2**). Using the Laplace’s equation approach, streamlines providing one-to-one correspondence without crossings between the periosteal and the endosteal surfaces were computed. The lengths of these streamlines represent the Surf.app.Ct.Th, while the integrals of the soft cortical bone classifications along the trajectories of the streamlines from the periosteal to the endosteal surfaces represent the Surf.Ct.SIT. The mean of BMD and SED values along the trajectories of the streamlines represent the Surf.Ct.BMD and Surf.Ct.SED, respectively.

The smooth voxel-based and the surface-based parametric maps described above were then spatially normalized to a common template of the distal radius as previously described (28). This spatial normalization step established anatomical correspondences across the parametric maps of all subjects in the study enabling the evaluation of voxel-wise and vertex-wise interrelationships of bone parameters. The voxel-based spatially-normalized maps were eroded to avoid the cortical bone and generate spatially-normalized maps of: 1) homogenized Tb.BMD (H.Tb.BMD), 2) homogenized Tb.1/N (H.Tb.1/N), 3) homogenized Tb.SED (H.Tb.SED), and 4) local Tb.BV/TV (L.Tb.BV/TV).

### Statistical Analysis

#### Standard Analysis

Mean values of parameters of bone density, microstructure, geometry and biomechanics were compared between controls and fracture cases with linear regression models adjusting for age, height, and weight.

For each compartment, within-group interrelationships of bone parameters were then evaluated using Spearman’s rank partial correlations adjusting for age, height and weight. These correlation coefficients were then compared between controls and fracture cases using the Fisher’s Z transformation.

*P*-values in these statistical tests were considered significant if they were less than 0.05.

#### Spatial Analysis

To assess within-group spatial interrelationships of bone parameters, trabecular voxel-wise and cortical vertex-wise Spearman’s rank partial correlations adjusted for age, height, weight, and shape (90% of variance = first 4 principal components) were computed using the spatially-normalized parametric maps. This step generated correlation coefficient maps and their corresponding *P*-value maps in the template space. Given the large number of correlations, *P*-value maps were corrected for multiple comparisons using the false discovery rate method (FDR; *q*=0.05) (30). Spatial comparisons of the correlation coefficients between controls and fracture cases were then performed with voxel-wise and vertex-wise Fisher’s Z transformations generating *P*-value maps that were also corrected for multiple comparisons using FDR (*q* = 0.05).

To better understand the spatial correlations, we also performed statistical analyses of the smooth voxel-based maps of trabecular parameters and the surface-based maps of cortical parameters with no spatial normalization. In particular, we: 1) compared mean parametric values between controls and cases using linear regression models adjusting for age, height, and weight; 2) performed within-group Spearman’s rank partial correlations of bone parameters within each compartment adjusting for age, height and weight; 3) performed within-group Spearman’s rank partial correlations of bone parameters with μFEA.FL within each compartment adjusting for age, height and weight; and 4) compared the correlation coefficients between controls and fracture cases using Fisher’s Z transformations. *P*-values in these statistical tests were considered significant if they were less than 0.05.

## Results

### Subject Characteristics

The characteristics of the subjects included in this study are shown in **Table 1**. Twenty-three scans out of 205 were excluded because of severe motion artifacts (grade > 3). Therefore, 182 post-menopausal women were included in the analysis: 98 controls and 84 with a newly diagnosed Colles’ fracture. Controls and fracture cases showed no significant differences in age, height, and weight (all *p* > 0.05).

**Table 1:** Subject characteristics.

	Controls	Fracture cases	*p*-value
Number	98	84	
Age (years)	65.3 ± 9.3	63.8 ± 9.3	NS
Height (cm)	161.2 ± 5.6	161.8 ± 5.7	NS
Weight (kg)	73.6 ± 13.8	74.4 ± 17.7	NS

Values are shown as mean ± standard deviation.

Two-sample t-test.

NS = P ≥ 0.05.

### Standard Analysis

#### Global Comparisons

The differences between groups in BMD, bone microstructure, geometry and μFEA parameters are summarized in **Table 2**. Subjects with fracture had significantly lower Tb.BMD, Tb.BV/TV, Tb.N, Ct.BMD and Ct.Th (all *p* < 0.01), and significantly higher Tb.Sp (*p* = 0.015) than controls. In addition, the fracture cases had significantly lower μFEA.FL and μFEA.Stiffness than controls (both *p* < 0.001).

**Table 2:** Bone parameters assessed with the standard analysis method.

Parameter (units)	Controls	Fracture cases	*p*-value
Tb.BMD (mg/cm^3^)	144.6 ± 39.6	121.1 ± 39.3	<0.001*
Tb.BV/TV	0.23 ± 0.05	0.19 ± 0.05	<0.001*
Tb.N (1/mm)	1.67 ± 0.36	1.47 ± 0.38	<0.001*
Tb.Sp (mm)	0.64 ± 0.30	0.74 ± 0.26	0.015*
Ct.BMD (mg/cm^3^)	886.0 ± 73.0	858.4 ± 76.1	<0.001*
Ct.Th (mm)	1.01 ± 0.22	0.95 ± 0.19	<0.01*
μFEA.FL (N)	2539.3 ± 467.1	2285.0 ± 492.9	<0.001*
μFEA.Stiffness (N/mm)	44070.2 ± 8425.7	39799.0 ± 8675.9	<0.001*

Values are shown as mean ± standard deviation.

Linear regression adjusting for age, height, and weight.

*Significant at p < 0.05.

#### Global Correlations

All the interrelationships of trabecular and cortical bone parameters were significant within each group (*p* < 0.001), but only the interrelationship of Tb.BMD-Tb.N was significantly different between controls and fracture cases (*p* < 0.05) as shown in **Table 3**.

**Table 3:** Global compartmental Spearman’s rank partial correlations for bone parameters assessed with the standard analysis method.

Interrelationships		Controls		Fracture cases		Controls vs. Fracture cases
		ρ	*p*-value	ρ	*p*-value	*p*-value
Tb.BMD	Tb.BV/TV	0.98	<0.001*	0.99	<0.001*	NS
Tb.BMD	Tb.N	0.83	<0.001*	0.91	<0.001*	0.03*
Tb.BMD	Tb.Sp	-0.86	<0.001*	-0.91	<0.001*	NS
Tb.BMD	μFEA.FL	0.67	<0.001*	0.65	<0.001*	NS
Tb.BMD	μFEA.Stiffness	0.62	<0.001*	0.60	<0.001*	NS
Tb.BV/TV	Tb.N	0.85	<0.001*	0.91	<0.001*	NS
Tb.BV/TV	Tb.Sp	-0.88	<0.001*	-0.92	<0.001*	NS
Tb.BV/TV	μFEA.FL	0.65	<0.001*	0.65	<0.001*	NS
Tb.BV/TV	μFEA.Stiffness	0.58	<0.001*	0.59	<0.001*	NS
Tb.N	Tb.Sp	-0.99	<0.001*	-0.99	<0.001*	NS
Tb.N	μFEA.FL	0.48	<0.001*	0.59	<0.001*	NS
Tb.N	μFEA.Stiffness	0.40	<0.001*	0.54	<0.001*	NS
Tb.Sp	μFEA.FL	-0.51	<0.001*	-0.59	<0.001*	NS
Tb.Sp	μFEA.Stiffness	-0.43	<0.001*	-0.55	<0.001*	NS
μFEA.FL	μFEA.Stiffness	0.99	<0.001*	0.99	<0.001*	NS
Ct.BMD	Ct.Th	0.69	<0.001*	0.63	<0.001*	NS

ρ = Spearman’s rank partial correlation coefficient.

Spearman’s rank partial correlations were adjusted for age, height and weight.

The Fisher’s Z transformation was used to compare correlation coefficients between groups.

*Significant at p < 0.05.

NS = P ≥ 0.05.

### Spatial Analysis

#### Statistical Maps

**Figure 3:**
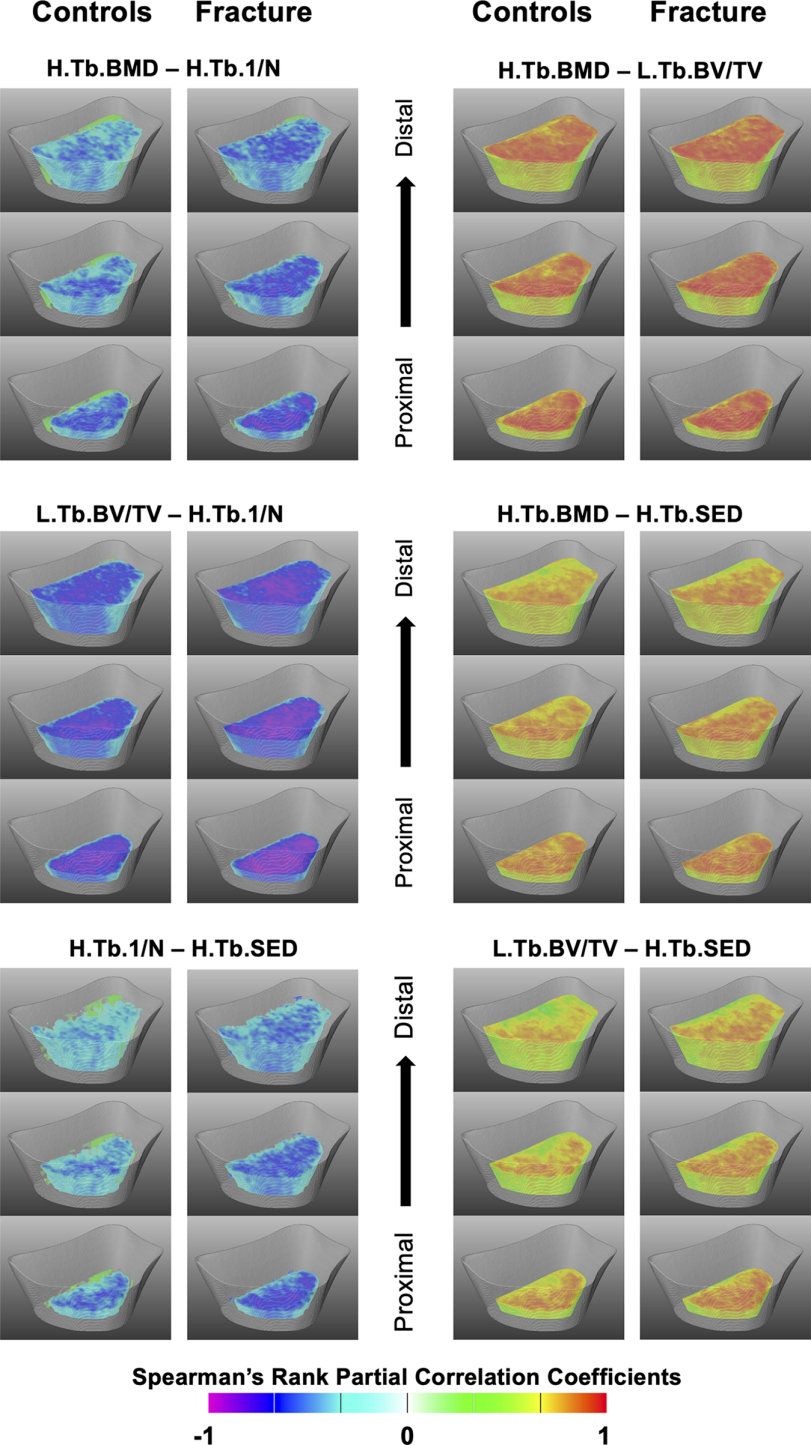
Spatial assessment of interrelationships of trabecular bone parameters. Three-dimensional views at three different levels (1/4, ½ and ¾ of the scan length) of the statistical maps showing Spearman’s rank partial correlation coefficients for the different interrelationships of trabecular bone parameters for controls and fracture cases. All maps were adjusted for age, height, weight, and shape. Voxels that were no significant after correcting for multiple comparisons were rendered transparent.

**Figure 4:**
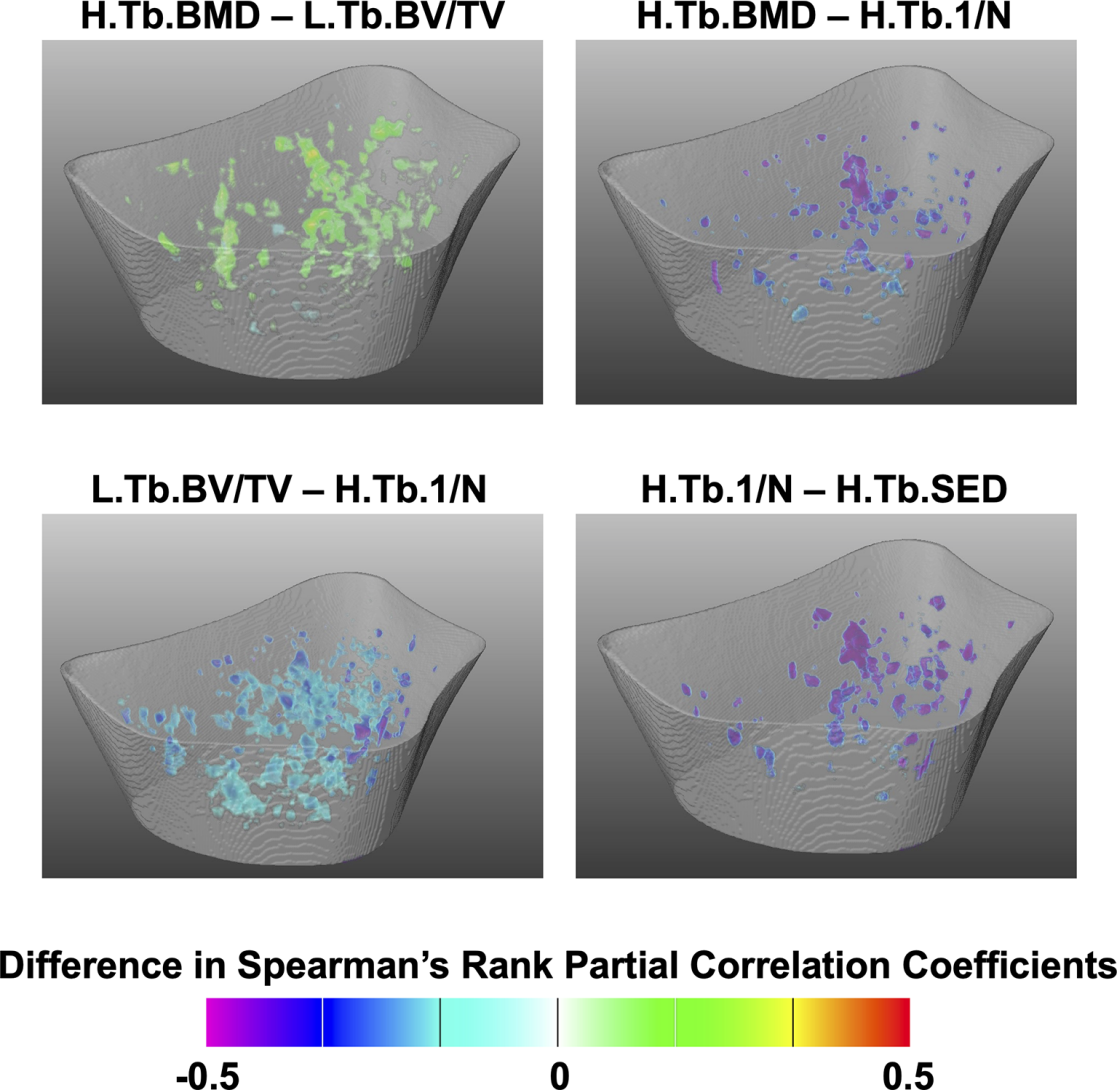
Assessment of spatial differences of interrelationships of trabecular bone parameters between controls and fracture cases. Three-dimensional statistical maps showing voxels for which the Spearman’s rank partial correlation coefficients were significantly different between fracture cases and controls. Significant voxels are showing the difference of correlation coefficients (fracture cases minus controls), and nonsignificant voxels were rendered transparent.

The Spearman’s rank partial correlation coefficient maps for the different interrelationships of trabecular bone parameters are shown in **Figure 3** for both controls and fracture cases. These maps revealed significant heterogeneous spatial distributions of correlation coefficients across the distal radius, particularly with stronger correlations proximally than distally in both groups. In addition, in most of the correlation maps, fracture cases showed stronger correlations than controls, which was manifested with clusters of significant different correlation coefficients as shown in **Figure 4** for H.Tb.BMD–L.Tb.BV/TV, H.Tb.BMD–H.Tb.1/N, L.Tb.BV/TV–H.Tb.1/N, and H.Tb.1/N–H.Tb.SED.

**Figure 5:**
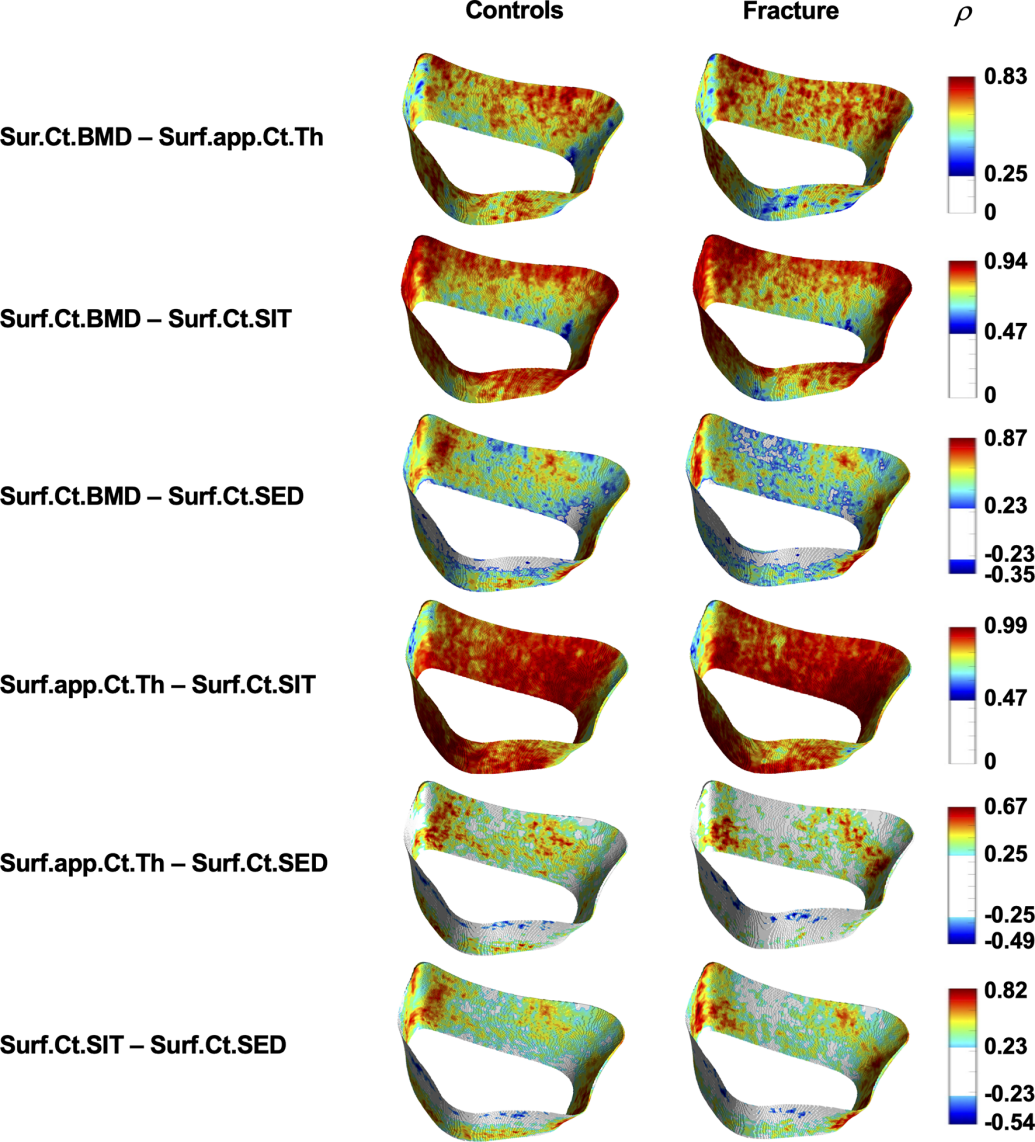
Spatial assessment of interrelationships of cortical bone parameters. Surface statistical maps showing Spearman’s rank partial correlation coefficients for the different interrelationships of cortical bone parameters for controls and fracture cases. All maps were adjusted for age, height, weight, and shape. Vertices that were no significant after correcting for multiple comparisons are shown in white. *ρ* = Spearman’s rank partial correlation coefficient.

**Figure 6:**
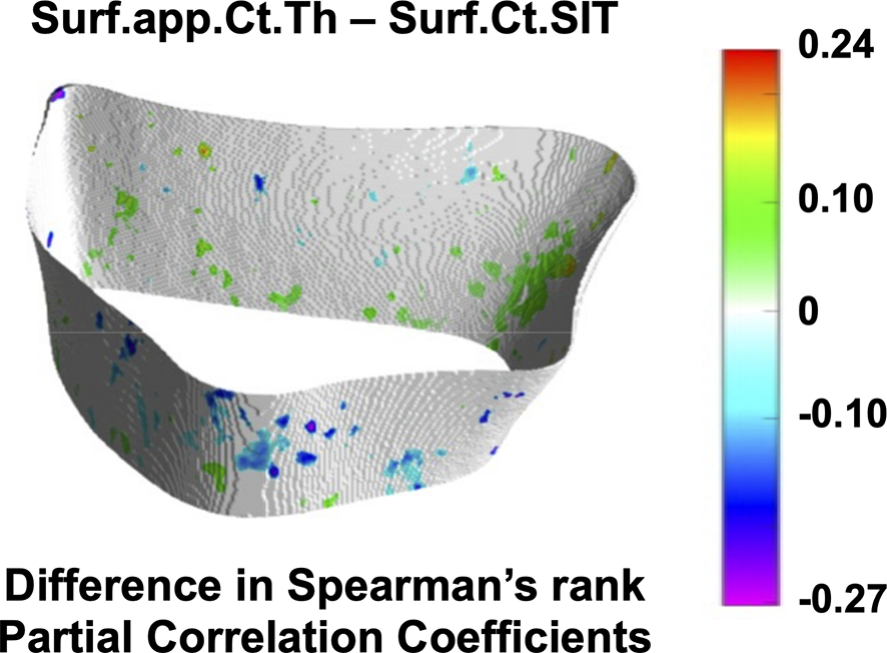
Assessment of spatial differences of interrelationships of cortical bone parameters between controls and fracture cases. Surface statistical map showing vertices for which the Spearman’s rank partial correlation coefficients were significantly different between controls and fracture cases for the interrelationship of Surf.app.Ct.Th–Surf.Ct.SIT. Significant vertices are showing the difference of correlation coefficients (fracture cases minus controls) with nonsignificant vertices displayed in white. Surf.app.Ct.Th–Surf.Ct.SIT was the only cortical bone interrelationship that showed significant spatial differences between controls and fracture cases.

Cortical Spearman’s rank partial correlation coefficient maps are depicted in **Figure 5** for both controls and fracture cases. These maps also revealed significant heterogeneous distributions of correlation coefficients across the distal radius within each group. However, these heterogeneous distributions were not significantly different between controls and fracture cases, except for the correlation of Suf.app.Ct.Th–Surf.Ct.SIT as is shown in **Figure 6**.

#### Global Comparisons

Differences in homogenized and local trabecular voxel-based parameters, and in cortical surface-based parameters with no spatial normalization are summarized in **Table 4**. Fracture cases had significantly lower H.Tb.BMD, L.Tb.BV/TV, H.Tb.SED, Surf.Ct.BMD, Surf.app.Ct.Th and Surf.Ct.SIT than controls (all *p* < 0.001). Fracture cases also showed significantly higher H.Tb.1/N than controls (*p* < 0.05). However, Surf.Ct.SED did not differ between groups.

**Table 4:** Homogenized and local trabecular voxel-based parameters, and cortical surface-based parameters with no spatial normalization.

Parameter	Controls	Fracture cases	*p*-values
H.Tb.BMD (mg/cm^3^)	354.2 ± 37.0	332.5 ± 35.5	<0.001*
L.Tb.BV/TV	0.22 ± 0.05	0.19 ± 0.05	<0.001*
H.Tb.1/N (voxels)	7.92 ± 3.40	8.91 ± 2.87	0.020*
H.Tb.SED (J/mm^3^)	0.08 ± 0.03	0.06 ± 0.03	<0.001*
Surf.Ct.BMD (mg/cm^3^)	830.6 ± 91.5	793.5 ± 91.2	<0.001*
Surf.app.Ct.Th (mm)	0.98 ± 0.17	0.91 ± 0.15	<0.001*
Surf.Ct.SIT (mm)	0.72 ± 0.14	0.66 ± 0.12	<0.001*
Surf.Ct.SED (J/mm^3^)	0.27 ± 0.03	0.26 ± 0.03	NS

Values are shown as mean ± standard deviation.

Linear regression adjusting for age, height, and weight.

*Significant at p < 0.05.

NS = P ≥ 0.05.

#### Global Correlations

The interrelationships of homogenized and local trabecular voxel-based parameters, and cortical surface-based parameters with no spatial normalization using Spearman’s rank partial correlations are summarized in **Table 5**. With the exception of the correlation of H.Tb.1/N– H.Tb.SED for controls (*p* = 0.006), all within-group compartmental trabecular and cortical Spearman’s rank partial correlations were significant at *p* < 0.001. In addition, the correlations of H.Tb.BMD–L.Tb.BV/TV, H.Tb.BMD–H.Tb.1/N, L.Tb.BV/TV–H.Tb.1/N, Surf.Ct.BMD–Surf.Ct.SED, and Surf.Ct.SIT–Surf.Ct.SED were significantly different between groups at *p* < 0.05.

**Table 5:** Global compartmental Spearman’s rank partial correlations for homogenized and local trabecular voxel-based parameters, and for cortical surface-based parameters with no spatial normalization.

Interrelationships		Controls		Fracture cases		Controls vs. Fracture cases
		ρ	*p*-value	ρ	*p*-value	*p*-value
H.Tb.BMD	L.Tb.BV/TV	0.83	<0.001*	0.93	<0.001*	0.003*
H.Tb.BMD	H.Tb.1/N	-0.44	<0.001*	-0.75	<0.001*	0.001*
H.Tb.BMD	H.Tb.SED	0.78	<0.001*	0.65	<0.001*	NS
L.Tb.BV/TV	H.Tb.1/N	-0.82	<0.001*	-0.91	<0.001*	0.015*
L.Tb.BV/TV	H.Tb.SED	0.64	<0.001*	0.62	<0.001*	NS
H.Tb.1/N	H.Tb.SED	-0.28	0.006*	-0.44	<0.001*	NS
Surf.Ct.BMD	Surf.app.Ct.Th	0.82	<0.001*	0.77	<0.001*	NS
Surf.Ct.BMD	Surf.Ct.SIT	0.86	<0.001*	0.83	<0.001*	NS
Surf.Ct.BMD	Surf.Ct.SED	0.80	<0.001*	0.59	<0.001*	0.004*
Surf.app.Ct.Th	Surf.Ct.SIT	0.99	<0.001*	0.99	<0.001*	NS
Surf.app.Ct.Th	Surf.Ct.SED	0.61	<0.001*	0.40	<0.001*	NS
Surf.Ct.SIT	Surf.Ct.SED	0.66	<0.001*	0.45	<0.001*	0.046*

ρ = Spearman’s rank partial correlation coefficient.

Spearman’s rank partial correlations were adjusted for age, height and weight.

The Fisher’s Z transformation was used to compare correlation coefficients between groups.

*Significant at p < 0.05.

NS = P ≥ 0.05.

In terms of correlations with μFEA.FL, mean values of the homogenized and local trabecular voxel-based parameters and cortical surface-based parameters manifested significant correlations in both groups, except for Ct.SED in the fracture group. There were no significant differences in the strength of these correlations between groups (**Table 6**).

**Table 6:** Global compartmental Spearman’s rank partial correlations of μFEA.FL with homogenized and local trabecular voxel-based parameters, and cortical surface-based parameters with no spatial normalization.

Interrelationships	Controls		Fracture cases		Controls vs. Fracture cases
	ρ	*p*-value	ρ	*p*-value	*p*-value
H.Tb.BMD	0.76	<0.001*	0.68	<0.001*	NS
L.Tb.BV/TV	0.71	<0.001*	0.67	<0.001*	NS
H.Tb.1/N	-0.45	<0.001*	-0.59	<0.001*	NS
H.Tb.SED	0.67	<0.001*	0.57	<0.001*	NS
Surf.Ct.BMD	0.52	<0.001*	0.46	<0.001*	NS
Surf.app.Ct.Th	0.69	<0.001*	0.75	<0.001*	NS
Surf.Ct.SIT	0.68	<0.001*	0.72	<0.001*	NS
Surf.Ct.SED	0.28	0.005*	0.17	NS	NS

ρ = Spearman’s rank partial correlation coefficient.

Spearman’s rank partial correlations were adjusted for age, height and weight.

The Fisher’s Z transformation was used to compare correlation coefficients between groups.

*Significant at p < 0.05.

NS = P ≥ 0.05.

## Discussion

In this study, we assessed the spatial distribution of interrelationships of parameters of bone density, microstructure, geometry and biomechanics in the distal radius of postmenopausal women with and without a recent Colles’ fracture using HR-pQCT. Spearman’s rank partial correlation coefficient maps showed significant heterogeneous spatial distributions of these interrelationships across the distal radius for both groups. In addition, small clusters of significant different interrelationships between controls and fracture cases were identified, particularly for trabecular bone, with stronger interrelationships in the fracture cases.

As previously reported by Melton et al. (18) and in agreement with previous cross-sectional studies (12, 16, 19), we found that the fracture cases had lower BMD, deterioration of bone microstructure and geometry, and lower μFEA-derived bone strength compared with controls using the standard analysis techniques provided by the manufacturer (**Table 2**). Similar trends were observed for the global analyses of homogenized and local trabecular bone parameters, as well as for the surface-based assessments of cortical bone with no spatial normalization (**Table 4**). However, in contrast to H.Tb.SED, for which fracture cases showed significantly lower values than controls, Surf.Ct.SED was not significantly different between the two groups. This result is consistent with the original study of Melton et al. using the standard analysis method (18). SED represents the area under the stress-strain curve (31, 32) and reflects the local energy stored per unit volume under the apparent load; therefore, our results suggest that the resistance to fracture in the cortical bone was maintained to some extent even in the fracture group. The lack of a significant difference in Surf.Ct.SED may be explained by compensatory prevention mechanisms of cortical deterioration (16) or to the axial load configuration of the μFEA, which does not fully resemble fall-loading conditions. This result may be also partly attributed to the fact that the radius is an unloaded site.

Despite global differences in trabecular and cortical bone parameters between controls and fracture cases, results showed expected global significant correlations of bone density, microstructure, geometry and biomechanics for both the trabecular and the cortical compartments within each group, for both analysis methods (**Tables 3** and **5**). Our results are in agreement with those of Boutroy et al., who also reported significant expected interrelationships between density and microstructural parameters at the distal radius in postmenopausal women using HR-pQCT (15). In that study, the trabecular correlations were in the absolute range of 0.78–0.91, while the correlation of Ct.BMD with Ct.Th was equal to 0.95. Here, the trabecular correlations between density and microstructural parameters were in the absolute range of 0.83–0.99, with the correlation of Ct.BMD and Ct.Th equal to 0.69 and 0.63 for controls and fracture cases, respectively. Our results also demonstrated significant correlations of density, microstructure and geometry with biomechanical parameters derived from μFEA (|*ρ*| = 0.40–0.67). Moreover, we identified significant heterogeneous spatial distributions of interrelationships of bone parameters across the distal radius for both the trabecular (**Figure 3**) and the cortical bone compartments (**Figure 5**) within each group. In particular, both groups manifested stronger Spearman’s rank partial correlations proximally than distally for most of the trabecular interrelationships; with an opposite trend for the cortical interrelationships of Surf.Ct.BMD with Surf.app.Ct.Th and Surf.Ct.SIT, i.e., stronger correlations distally than proximally for both groups. These observations might be partly explained by the study of Boyd (33), who showed an increase in Tb.N and a decrease in Ct.Th from the proximal to the distal end of the radius. Interestingly, the local interrelationships of Surf.Ct.SED with other cortical parameters were in general small, even to the point of showing nonsignificant areas on the distal end for both groups. Another interesting finding for SED, was that for the trabecular compartment the correlations were in general stronger in the dorsal side, while for the cortical compartment they were stronger in the palmar side in both groups. Although previous studies have demonstrated regional structural variations of trabecular and cortical bone (34, 35), the regions in those studies were predefined, limiting the local assessments to the size and location of those regions. Here, by using our statistical parametric mapping (SPM) framework for HR-pQCT studies (28), comprehensive local assessments of interrelationships of bone parameters were possible across the distal radius in both the trabecular and cortical compartments. Nevertheless, results of this study and those based on predefined regions of interest (34–36) underscore the relevance of local assessments parallel to the global analyses to improve our understanding of bone strength.

Although we identified significant global and local interrelationships between all bone parameters within each group, and even similar heterogeneous spatial distributions of the correlation coefficients between groups, the results showed significant different global (**Tables 3** and **5**) and local interrelationships (**Figures 4** and **6**) between groups. In terms of cortical bone, there were no significant differences between groups using the standard analysis techniques (**Table 3**). However, the compartmental interrelationships of Surf.Ct.SED with Surf.Ct.BMD and Surf.Ct.SIT showed significant differences between groups using surface-based assessments (**Table 5**). In addition, the Surf.app.Ct.Th–Surf.Ct.SIT partial correlation coefficient maps yielded small clusters of significant correlation differences between groups (**Figure 6**). The differences in the compartmental interrelationships of Surf.Ct.SED might be indicators of compensatory mechanisms in the cortical bone to maintain bone strength in the fracture group. The spatial differences in the Surf.app.Ct.Th–Surf.Ct.SIT correlations between groups were probably due to cortical porosity which is taken into account by Surf.Ct.SIT. In terms of trabecular bone, the partial correlations of H.Tb.BMD–L.Tb.BV/TV, H.Tb.BMD–H.Tb.1/N, and L.Tb.BV/TV–H.Tb.1/N were significantly different between groups both at the global (**Table 5**) and local level (**Figure 4**), although only the interrelationship of Tb.BMD–Tb.N was significantly different between groups using the standard analysis method (**Table 3**). In addition, small clusters of significant correlation differences between groups were observed for H.Tb.1/N–H.Tb.SED (**Figure 4**). This correlation was not significantly different between groups at the compartmental level (**Table 5**). An interesting finding was that in the significant global and local correlation differences in the trabecular bone, fracture cases showed stronger correlation coefficients than controls. The opposite effect was observed for cortical bone, i.e., in the significant global and local correlation differences, controls showed stronger correlation coefficients than the fracture cases. These compartmental differences in the strength of the correlations between controls and fracture cases indicate that postmenopausal women with a recent Colles’ fracture might undergo a synergistic decline in bone quantity and quality in the trabecular bone compartment, with a differential response of bone properties in the cortical compartment, effectively affecting compartmental bone biomechanics as was observed with SED (**Table 4**).

In agreement with previous studies, our results suggest that assessment of the trabecular bone in the distal radius might yield parameters that are more sensitive to prevent fragility fractures (6, 7), including Colles’ fracture (16). In fact, a previous HR-pQCT study demonstrated that trabecular bone microstructure at baseline is important to predict the risk of fracture in postmenopausal women with denosumab treatment (37). Therefore, our results may indicate that the loss of both BMD and bone quality on the trabecular compartment is likely to be strongly associated with fragility fractures.

This study has two main limitations. First, Colles’ fractures occurred with a median of 7 months prior to the HR-pQCT scans, i.e., this is not a prospective study, which limits to certain extent the impact of our findings. Second, the unaffected side had to be scanned for the fracture group, which might not fully represent the affected side since a previous study demonstrated side-to-side differences of cortical area and failure load at the radius of healthy women (38). However, in the same study, no significant differences regarding other cortical and trabecular parameters were reported.

In conclusion, we assessed global and spatial interrelationships of bone density, microstructure, geometry, and biomechanics in postmenopausal women with and without a Colles’ fracture, as well as the global and spatial differences of these interrelationships between the control and fracture groups. We showed significant heterogeneous spatial distributions of these interrelationships across the distal radius within each group, and also found small clusters of significant differences for these interrelationships between groups, particularly in the trabecular bone compartment. Our findings indicate that local bone properties of BMD, microstructure, and geometry are heterogeneous in the distal radius, and that trabecular bone parameters may play a major role in the assessment of bone fragility at this anatomical site.

## Data Availability Statement

The data analyzed in this study is subject to the following licenses/restrictions: The HR-pQCT images are owned by Mayo Clinic. Requests to access these datasets should be directed to principal investigator SK.

## Author Contributions

KS: Analysis and interpretation of data, manuscript drafting, and revision. AB: Conception and design, interpretation of data, and manuscript revision. MO: Interpretation of data and manuscript revision. SK: Principal investigator of the original Colles’ fracture study, interpretation of data, and manuscript revision. JC-G: Conception and design, analysis and interpretation of data, manuscript drafting and revision. All authors contributed to the article and approved the submitted version.

## Funding

This work was supported in part by NIH R01AR068456 and NIH R01AR027065.

## Conflict of Interest

The authors declare that the research was conducted in the absence of any commercial or financial relationships that could be construed as a potential conflict of interest.
